# Proteomic analysis reveals inhibition of mevalonate and glycolysis pathways in hepatocytes by 27-hydroxycholesterol

**DOI:** 10.1042/BCJ20253035

**Published:** 2025-08-04

**Authors:** Wan-Seog Shim, Seulah Lee, Bakhovuddin Azamov, Chanhee Lee, Yeowon Kang, Kwang Min Lee, Changwan Hong, Sang-Mo Kwon, Koanhoi Kim, Dongjun Lee, Jong Hyuk Yoon, Parkyong Song

**Affiliations:** 1 Department of Convergence Medicine, Pusan National University School of Medicine, Yangsan, 50612, Republic of Korea; 2 Neurodegenerative Diseases Research Group, Korea Brain Research Institute, Daegu, 41062, Republic of Korea; 3 Department of Life Science and Environmental Biochemistry, Pusan National University, Miryang, 50463, Republic of Korea; 4 Department of Anatomy, Pusan National University School of Medicine, Yangsan, 50612, Republic of Korea; 5 Department of Convergence Medical Science, Pusan National University School of Medicine, Yangsan, 50612, Republic of Korea; 6 Laboratory for Vascular Medicine and Stem Cell Biology, Department of Physiology, Medical Research Institute, School of Medicine, Pusan National University, Yangsan, 50612, Republic of Korea; 7 Department of Pharmacology, Pusan National University School of Medicine, Yangsan, 50612, Republic of Korea

**Keywords:** 27-hydroxycholesterol, glycolysis, mevalonate pathway, Nrf2/HO-1 signaling, proteomics

## Abstract

27-Hydroxycholesterol (27OHC), an endogenous oxysterol, has been implicated in various physiological processes, including regulation of estrogen receptor activity and lipid metabolism. However, studies on how 27OHC affects the metabolic changes associated with lipogenesis inhibition in the liver remain limited. This study aimed to investigate the systemic effects of 27OHC on hepatocytes through a comparative proteomic analysis of the proteomes in the 27OHC-treated *Mus musculus* hepatocyte (AML12) cells. Ingenuity Pathway Analysis revealed significant down-regulation of certain metabolic pathways, such as cholesterol biosynthesis and glycolysis, which are highly associated with lipid metabolism, following 27OHC treatment. Furthermore, *in vitro* biochemical analysis revealed significant inhibition of the expression of genes associated with the mevalonate (MVA) pathway and a decrease in the total cellular cholesterol levels in AML12 cells and primary hepatocytes following 27OHC treatment. In addition, it was observed that 27OHC significantly reduced the transcript levels of critical glycolytic enzymes such as aldolase, phosphofructokinase, and pyruvate kinase. This inhibition resulted in decreased lactate production and extracellular acidification rate, indicating suppression of glycolytic flux. Concurrently, we proved that down-regulation of reactive oxygen species generation and hypoxia-inducible factor 1-alpha (HIF-1α) expression following 27OHC treatment partially contributed to glycolysis inhibition. Overall, we demonstrated the inhibitory effects of 27OHC on the hepatic MVA pathway and glycolysis, revealing a novel mechanism by which 27OHC regulates lipid metabolism. As the accumulation of cholesterol and lipids promotes hepatic fatty liver disease and increased glycolysis contributes to triacylglycerol maturation, the suppressive effects of 27OHC on hepatic lipid and glucose metabolism may contribute to protection against fatty liver development.

## Introduction

Oxysterols are oxygenated derivatives of cholesterol, formed either enzymatically or non-enzymatically through various biological processes [1]. These oxidized sterols can be produced enzymatically by specific enzymes, such as cytochrome P450 family members, or non-enzymatically by reactive oxygen species (ROS) and free radicals [2]. Notably, 25-hydroxycholesterol (25OHC), 27-hydroxycholesterol (27OHC), and 24S-hydroxycholesterol (24SOHC) are the common oxysterols. These oxysterols exhibit unique properties and functions influenced by a specific oxidation site [3]. The most abundant oxysterol in human circulation is typically 27OHC. This oxysterol is formed enzymatically by sterol 27-hydroxylase (CYP27A1), which is present in various tissues, including the liver, macrophages, and endothelial cells [4,5]. 27OHC has several intriguing biological functions, including acting as a selective estrogen receptor modulator (SERM). It selectively interacts with the estrogen receptor alpha (ERα) and acts as an ERα agonist in breast tissue, thus promoting proliferation and metastasis of ER-positive breast cancer cells [6,7]. These studies have revealed impaired growth of mammary tumors in *Cyp27a1^−/−^* mice, which lack 27OHC. Meanwhile, 27OHC acts as a competitive antagonist of the estrogen receptor in the vasculature [8]. Although estrogens generally exhibit protective cardiovascular effects, the function of 27OHC as a SERM may counteract these benefits under certain conditions, contributing to plaque formation and vascular inflammation [9]. Notably, its dual role as an agonist and antagonist, depending on the tissue type, underscores its complex function [10] and highlights its demand for targeted therapeutic strategies to mitigate its adverse effects while leveraging its beneficial actions.

In the context of lipid metabolism, 27OHC can exhibit complex effects on triglyceride (TAG) levels in metabolic tissues. Experimental studies using 3T3-L1 cells have demonstrated that 27OHC treatment can lead to a reduction in TAG content [11,12]. During adipogenic differentiation, 27OHC significantly reduces T0901317-mediated intracellular TAG accumulation. Mechanistically, 27OHC disrupts the adipogenic pathway, thus reducing PPARγ and aP2 expression and resulting in decreased synthesis and storage of TAGs [12]. Notably, reduced systemic lipid levels and improved insulin sensitivity were reported in transgenic mice overexpressing CYP27A1, the enzyme responsible for converting cholesterol into 27OHC [13]. These mice typically exhibited lower TAG levels and better glucose tolerance tests than wildtype controls. Moreover, 27OHC exhibits beneficial effects in reducing steatohepatitis, a form of liver inflammation and fat accumulation associated with non-alcoholic fatty liver disease [14]. Administration of 27OHC to *Ldlr^−/−^* mice or injecting bone marrow cells from mice overexpressing CYP27A1 suppressed the accumulation of lysosomal cholesterol and hepatic inflammation following a high-fat, high-cholesterol diet [14,15]. In contrast, a recent study reported that CYP7B1 activity, which is involved in 27OHC clearance, can attenuate metabolic-associated fatty liver disease (MAFLD) in mice, particularly in a thermoneutral condition. Although increased lipoprotein receptors in the livers of *Cyp7b1^−/−^* mice potentially alleviated MAFLD progression by enhancing the metabolism of lipotoxic molecules at lower temperatures (22°C), thermoneutral housing (30°C) exacerbated MAFLD more in CYP7B1-deficient mice, wherein higher 27OHC levels were present in circulation [16]. Therefore, 27OHC exemplifies the complexity of lipid metabolism and cellular regulation, exerting both beneficial and detrimental effects, depending on the context. Consequently, understanding the mechanisms through which 27OHC can mitigate adverse effects, along with enhancing its beneficial effects on hepatic lipid metabolism, is crucial.

Lipogenesis, the process of fatty acid synthesis, primarily occurs in the liver and adipose tissues. Several metabolic pathways contribute to lipogenesis [17]. Glycolysis is the metabolic pathway through which glucose is broken down into pyruvate. The pyruvate produced from glycolysis then enters the mitochondria and is converted to acetyl-CoA, a key building block for *de novo* lipogenesis (DNL) [18]. Notably, hepatic lipogenesis is tightly regulated by dietary nutrient composition and hormonal fluctuations. For instance, insulin promotes DNL by stimulating glycolysis and increasing expression of key lipogenic enzymes, such as acetyl-CoA carboxylase and fatty acid synthase (FAS) [19,20]. Importantly, NADPH is a crucial reducing agent required for the fatty acid synthesis process [21], and glycolysis contributes to NADPH production through the pentose phosphate pathway [22]. Therefore, increased glycolysis through high carbohydrate intake or hyperinsulinemia may contribute to abnormal lipid accumulation in the liver [23,24]. In addition to glucose metabolism, the cholesterol synthetic process, also known as the mevalonate (MVA) pathway, indirectly contributes to lipogenesis. Although the MVA pathway itself does not directly produce fatty acids, its downstream products play roles in lipid metabolism. For example, isoprenoids generated from the MVA pathway are essential for the formation of lipid rafts and post-translational modification of proteins, which are involved in signaling pathway for lipid synthesis [25,26]. Recently, Trub et al. reported that inhibition of the cholesterol biosynthetic pathway induced protein 3-hydroxyl-3-methylglutarylation (HMGylation) on FAS, leading to a reduction in the activity of FAS [27]. Because both cholesterol and fatty acids are essential components of lipid droplets in cells [28], the synthesis of cholesterol esters is crucial for energy storage as lipids. Additionally, several studies have shown that oxidative stress, a state of imbalance between ROS production and antioxidant defense, can affect lipid accumulation in adipose tissues [29,30] and increase glycolysis [31,32]. Although a strong association exists between lipogenesis inhibition and 27OHC, the potential effects of 27OHC on the hepatic MVA and glycolytic pathways, which can contribute to lipogenesis, remain unclear. Therefore, this study aimed to investigate the effects of 27OHC on these metabolic pathways in *Mus musculus* hepatocyte (AML12) cells and primary hepatocytes using proteomic and transcript analyses.

## Results

### Effect of 27OHC on hepatocytes' viability

**Figure 1:**
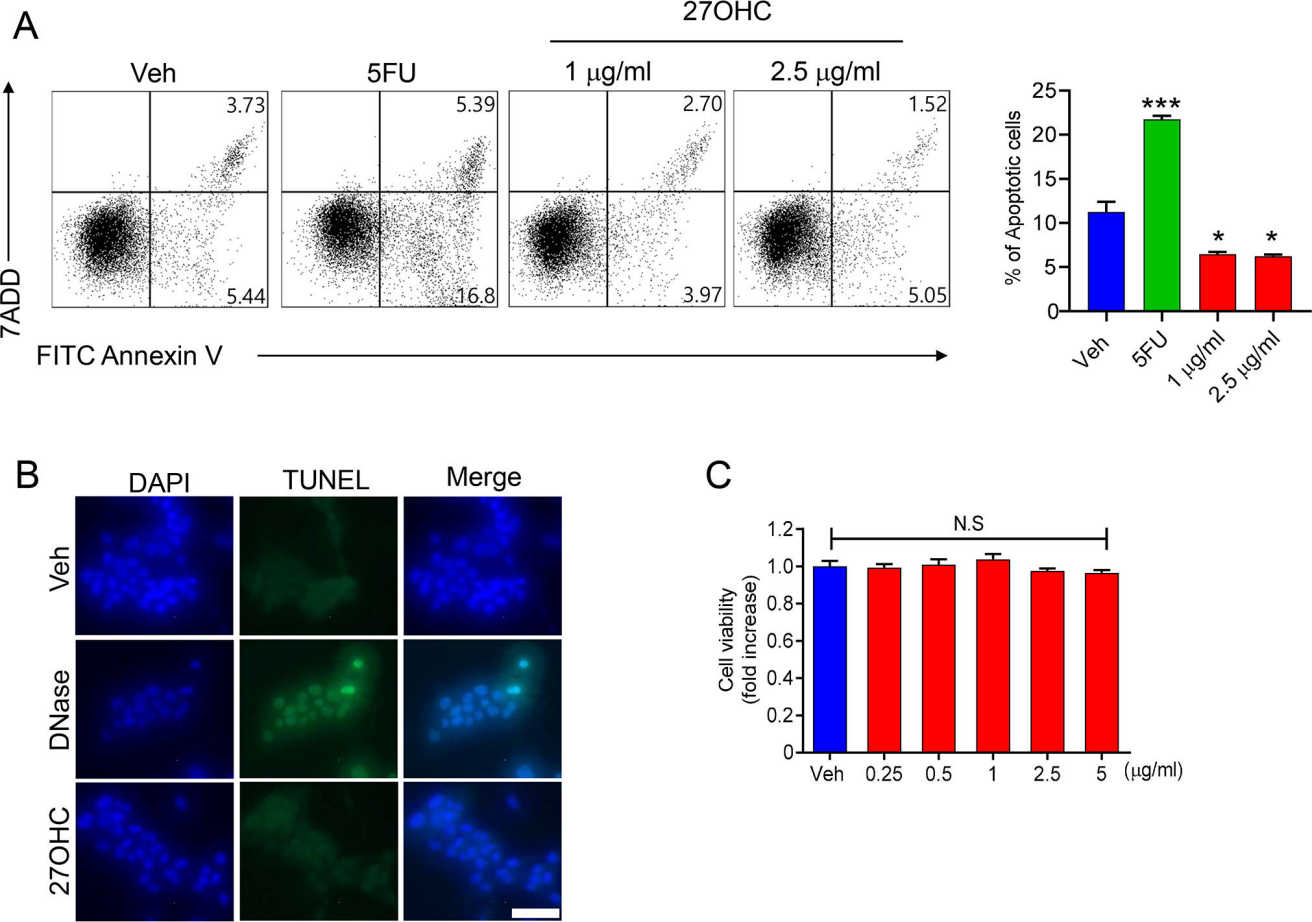
Cytotoxicity evaluation of 27-hydroxycholesterol in AML12 liver cells. (**A**) FACS plot showing frequency of apoptosis in AML12 cell populations following 5-FU (500 μM, positive control) and 27OHC (1, 2.5 μg/ml) treatment for 24 h. (**B**) TUNEL staining following 27OHC (2.5 μg/ml) treatment. DNase was used as a positive control. *n*=3 independent biological replicates for (**A, B**). (**C**) AML12 cells were incubated with different concentrations of 27OHC for 24 h. The cell viability was measured using the WST-1 assay (*n*=4). Statistical significance is from the pooled data of the multiple independent experiments. Values are presented as the mean ± S.E.M. *P<0.05 and ***P<0.001. N.S indicates not significant. 5-FU, 5-fluorouracil.

We evaluated the viability of hepatocytes using a fluorescence-activated cell sorting (FACS) assay to confirm whether 27OHC exerts cytotoxic effects on hepatocytes. AML12 cells were treated with the indicated concentrations of 27OHC for 24 h and then stained with 7-aminoactinomycin D (7-AAD) and Annexin V. Notably, the percentage of cells stained positive for apoptotic markers decreased following 27OHC treatment compared with the control groups. Furthermore, the proportion of 7-AAD-positive (necrotic) cells was comparable to that in the untreated controls, indicating no increase in necrotic populations (Figure 1A). In addition, fluorescence microscopy images showed comparable numbers of TUNEL-positive cells in the control and 27OHC groups (Figure 1B). Finally, the WST-1 assay further confirmed that the percentage of viable cells in the 27OHC-treated samples was statistically similar to that in the control samples (Figure 1C). Therefore, 27OHC treatment did not increase hepatocyte cell death.

### Comparative proteomics of AML12 cells following 27OHC treatment

**Figure 2:**
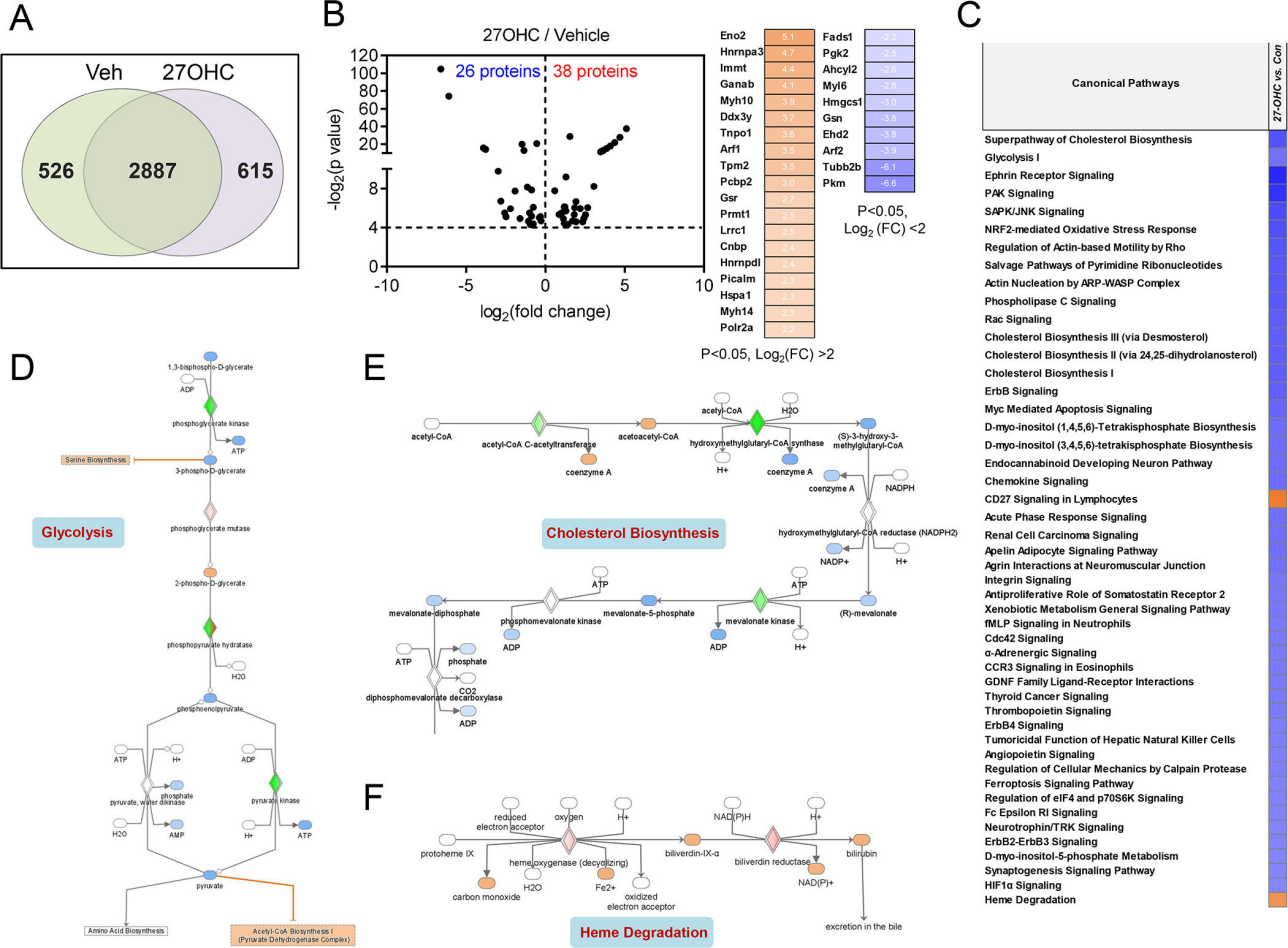
Quantitative proteomic analysis of 27-hydroxycholesterol-treated hepatocytes. (**A**) Venn diagram of the 4028 identified proteins. (**B**) Volcano plot (left) displays the overall proteins identified with a *P*-value of <0.05. Overall, 38 and 26 proteins were significantly up-regulated and down-regulated, respectively, following 27OHC (2.5 μg/ml) treatment (*n*=3). The right panel represents proteins with a *P*-value of <0.05 and a 2 log_2_ FC cutoff. (**C**) Comparative canonical pathway analyses using Ingenuity Pathway Analysis (IPA). Orange and blue indicate canonical pathways with a positive or negative Z‐score value of ≥2.0, respectively, for pathway activation. (**D**) IPA of glycolysis signaling in the proteome. (**E**) IPA of cholesterol biosynthesis signaling in the proteome. (**F**) IPA of heme degradation signaling in the proteome. Red and green indicate the increased and decreased protein levels, respectively. Orange and blue indicate the predicted activation and inhibition, respectively.

Global proteomics analysis was performed using a reliable quantitative LC–MS/MS approach. Proteomic analyses revealed 3413 and 3502 proteins in control and 27OHC-treated AML12 cells, respectively (Figure 2A). Notably, 526 and 615 proteins were exclusively identified in control and 27OHC-treated AML12 cells, respectively, with 2887 identified as common proteins (Figure 2B and Supplementary Table S1). Label-free quantitative analysis revealed significant differences in the levels of 64 proteins following 27OHC treatment (Table 1), which were visualized using the volcano plots (Figure 2B), wherein points above the non-axial horizontal line represented proteins with significantly different abundances (*P*<0.05). Notably, 26 and 38 proteins were down-regulated and up-regulated, respectively, following 27OHC treatment. The analysis of the significantly differentially expressed proteins (log_2_fold ≥2) in the proteome revealed the up-regulation of gamma-enolase (ENO2), heterogeneous nuclear ribonucleoprotein A3 (HNRNPA3), isoform 2 of MICOS complex subunit (IMMT), and down-regulation of acyl-CoA (8-3)-desaturase (FADS1), phosphoglycerate (PGK2), and putative adenosylhomocysteinase (AHCYL2).

**Table 1:** List of differentially expressed proteins by label-free quantitative analysis.

Symbol	Accession	Protein name	Total PSM		FC (Log2)	*P* value
			Vehicle	27OHC		
Eno2	P17183	Gamma-enolase	1.5	54.5	5.11	0.0000
Hnrnpa3	Q8BG05	Heterogeneous nuclear ribonucleoprotein A3	1.5	40.5	4.69	0.0000
Immt	Q8CAQ8-2	Isoform 2 of MICOS complex subunit Mic60	1.5	32.5	4.37	0.0000
Ganab	Q8BHN3-2	Isoform 2 of neutral alpha-glycosidase AB	1.5	27	4.10	0.0000
Myh10	Q61879	Myosin-10	1.5	23	3.87	0.0000
Ddx3y	Q62095	ATP-dependent RNA helicase DDX3Y	1.5	20	3.67	0.0001
Tnpo1	Q8BFY9	Transportin-1	1.5	19	3.59	0.0002
Arf1	P84078	ADP-ribosylation factor 1	1.5	18	3.52	0.0003
Tpm2	P58774	Tropomyosin beta chain	1.5	17.5	3.48	0.0004
Pcbp2	Q61990-2	Isoform 2 of poly(rC)-binding protein 2	1.5	13	3.05	0.0034
Gsr	P47791	Glutathione reductase, mitochondrial	1.5	10	2.67	0.0152
Prmt1	Q9JIF0-2	Isoform 2 of protein arginine N-methyltransferase 1	1.5	9	2.52	0.0252
Lrrc1	Q80VQ1	Leucine-rich repeat-containing protein 1	1.5	9	2.52	0.0252
Cnbp	P53996-3	Isoform 3 of cellular nucleic acid-binding protein	1.5	8.5	2.43	0.0324
Hnrnpdl	Q9Z130	Heterogeneous nuclear ribonucleoprotein D-like	1.5	8.5	2.43	0.0324
Picalm	Q7M6Y3-5	Phosphatidylinositol-binding clathrin assembly protein	1.5	8	2.35	0.0417
Hspa1a	Q61696	Heat shock 70 kDa protein 1A	1.5	8	2.35	0.0417
Myh14	Q6URW6	Myosin-14	1.5	8	2.35	0.0417
Polr2a	P08775	DNA-directed RNA polymerase II subunit RPB1	2.5	12	2.19	0.0162
Brix1	Q9DCA5	Ribosome biogenesis protein BRX1 homolog	4	16	1.93	0.0099
Mrpl28	Q9D1B9	39S ribosomal protein L28, mitochondrial	2.5	10	1.93	0.0415
Ubtf	P25976	Nucleolar transcription factor 1	2.5	10	1.93	0.0415
Syne2	Q6ZWQ0	Nesprin-2	4	15	1.84	0.0155
Mtx2	O88441	Metaxin-2	3.5	13	1.82	0.0249
Kpna1	Q60960	Importin subunit alpha-5	3.5	12	1.71	0.0388
Sptlc2	P97363	Serine palmitoyltransferase 2	3.5	12	1.71	0.0388
Tubb2a	Q7TMM9	Tubulin beta-2A chain	37	114	1.55	0.0000
Prpf6	Q91YR7	Pre-mRNA-processing factor 6	5	14	1.42	0.0498
Sorbs2	Q3UTJ2-2	Sorbin and SH3 domain-containing protein 2	16	41	1.29	0.0017
Arf4	P61750	ADP-ribosylation factor 4	9	23	1.29	0.0192
Ddx18	Q8K363	ATP-dependent RNA helicase DDX18	11	27	1.23	0.0143
Tpp2	Q64514-2	Isoform short of Tripeptidyl-peptidase 2	12	28.5	1.18	0.0146
Srp68	Q8BMA6	Signal recognition particle subunit SRP68	8	19	1.18	0.0461
Polr1c	P52432	DNA-directed RNA polymerases I and III subunit RPAC1	8	19	1.18	0.0461
Gsn	P13020	Gelsolin	10	23	1.13	0.0334
Myl6	Q60605	Myosin light polypeptide 6	14.5	31	1.03	0.0222
Tpr	F6ZDS4	Nucleoprotein TPR	21	40	0.86	0.0246
Tuba1a	P68369	Tubulin alpha-1A chain	76.5	121	0.59	0.0046
Eef1a1	P10126	Elongation factor 1-alpha 1	260	226	−0.27	0.039
Fasn	P19096	Fatty acid synthase	229	194	−0.31	0.029
Tubb4b	P68372	Tubulin beta-4B chain	150	121	−0.38	0.031
Flec1	Q9QXS1-13	Isoform PLEC-1F of plectin	409	292.5	−0.55	0.000
Gsr	P47791-2	Isoform cytoplasmic of glutathione reductase	35	21.5	−0.77	0.048
Tnpo1	Q8BFY9-2	Isoform 2 of Transportin-1	53	32.5	−0.77	0.015
Csde1	Q91W50	Cold shock domain-containing protein E1	35	20	−0.88	0.028
Cyp51a1	Q8K0C4	Lanosterol 14-alpha demethylase	58	33	−0.88	0.004
Fkbp5	Q64378	Peptidyl-prolyl cis-trans isomerase	24	13	−0.95	0.051
Eif4a2	P10630	Eukaryotic initiation factor 4A-II	30.5	16	−1.00	0.022
Mtpn	P62774	Myotrophin	22	11	−1.07	0.040
Fdps	Q920E5	Farnesyl pyrophosphate	40	19	−1.14	0.004
H2bc2	Q64524	Histone H2B type 2-E	53.5	22	−1.35	0.000
Eno3	P21550	Beta-enolase	77	29	−1.48	0.000
Idi1	P58044	Isopentenyl-diphosphate Delta-isomerase 1	13	4.5	−1.60	0.033
Pfkl	P12382	ATP-dependent 6-phosphofructokinase	18	5	−1.92	0.005
Fads1	Q920L1	Acyl-CoA (8-3)-desaturase	11	2.5	−2.21	0.016
Pgk2	P09041	Phosphoglycerate kinase 2	8	1.5	−2.48	0.029
Ahcyl2	Q68FL4	Putative adenosylhomocysteinase 3	8.5	1.5	−2.57	0.022
Myl6	Q60605-2	Isoform smooth muscle of myosin light polypeptide 6	10	1.5	−2.81	0.010
Hmgcs1	Q8JZK9	Hydroxymethylglutaryl-CoA synthase	15	2	−2.98	0.001
Gsn	P13020-2	Isoform 2 of gelsolin	19.5	1.5	−3.77	0.000
Ehd2	Q8BH64	EH domain-containing protein 2	20	1.5	−3.81	0.000
Arf2	Q8BSL7	ADP-ribosylation factor 2	21.5	1.5	−3.91	0.000
Tubb2b	Q9CWF2	Tubulin beta-2B chain	97.5	1.5	−6.09	0.000
Pkm	P52480-2	Isoform M1 of pyruvate kinase	137.5	1.5	−6.59	0.000

The Ingenuity Pathway Analysis (IPA) of the proteome dataset revealed that most (46/48) of the canonical pathways, including cholesterol biosynthesis, glycolysis, ephrin receptor signaling, and nuclear factor erythroid 2-related factor 2 (NRF2)-mediated oxidative stress response, with relatively high statistical significance, were deactivated following 27OHC treatment (Figure 2C). In contrast, the pathways such as CD27 signaling and heme degradation were activated following 27OHC treatment. We selected glycolysis signaling for subsequent canonical pathway analysis based on the observation that its regulatory pattern in the 27OHC proteome differed from that in the control proteome. Notably, the expression of 15 proteins (ALDOA, ENO1, ENO2, ENO3, GAPDH, GPI, PFKL, PFKM, PFKP, PGAM1, PGK1, PGK2, PKLR, PKM, and TPI1) in the glycolysis pathway differed between two proteomes and eventually induced deactivation of the pathway following 27OHC treatment (Figure 2D). In cholesterol biosynthesis signaling, the expression of 16 proteins (ACAA2, ACAT1, ACAT2, CYP51A1, EBP, FDPS, HADHA, HADHB, HMGCS1, IDI1, LBR, LSS, MSMO1, MVK, NSDHL, and SQLE) differed between two proteomes and eventually induced deactivation of the pathway (Figure 2E). Together with cholesterol metabolism, 27OHC treatment induced distinct changes in canonical pathways associated with lipid metabolism. In the context of fatty acid metabolism (Supplementary Figure 1A), 27OHC significantly activated β-oxidation pathways, as evidenced by positive Z-score values. Conversely, pathways involved in fatty acyl-CoA biosynthesis were down-regulated. Analysis of peroxisomal pathways revealed that 27OHC led to a reduction in class I peroxisomal membrane protein import pathway (Supplementary Figure 1B). However, peroxisomal lipid metabolism appeared largely unaffected in terms of overall activity, suggesting a selective impact of 27OHC on peroxisomal membrane composition rather than on general peroxisomal metabolic functions. Finally, heme degradation signaling was activated following 27OHC treatment because of the changes in the expression of four proteins (BLVRA, BLVRB, HMOX1, and HMOX2) between two proteomes (Figure 2F).

### 27OHC decreases cellular cholesterol levels by suppressing gene expression of the MVA pathway

**Figure 3:**
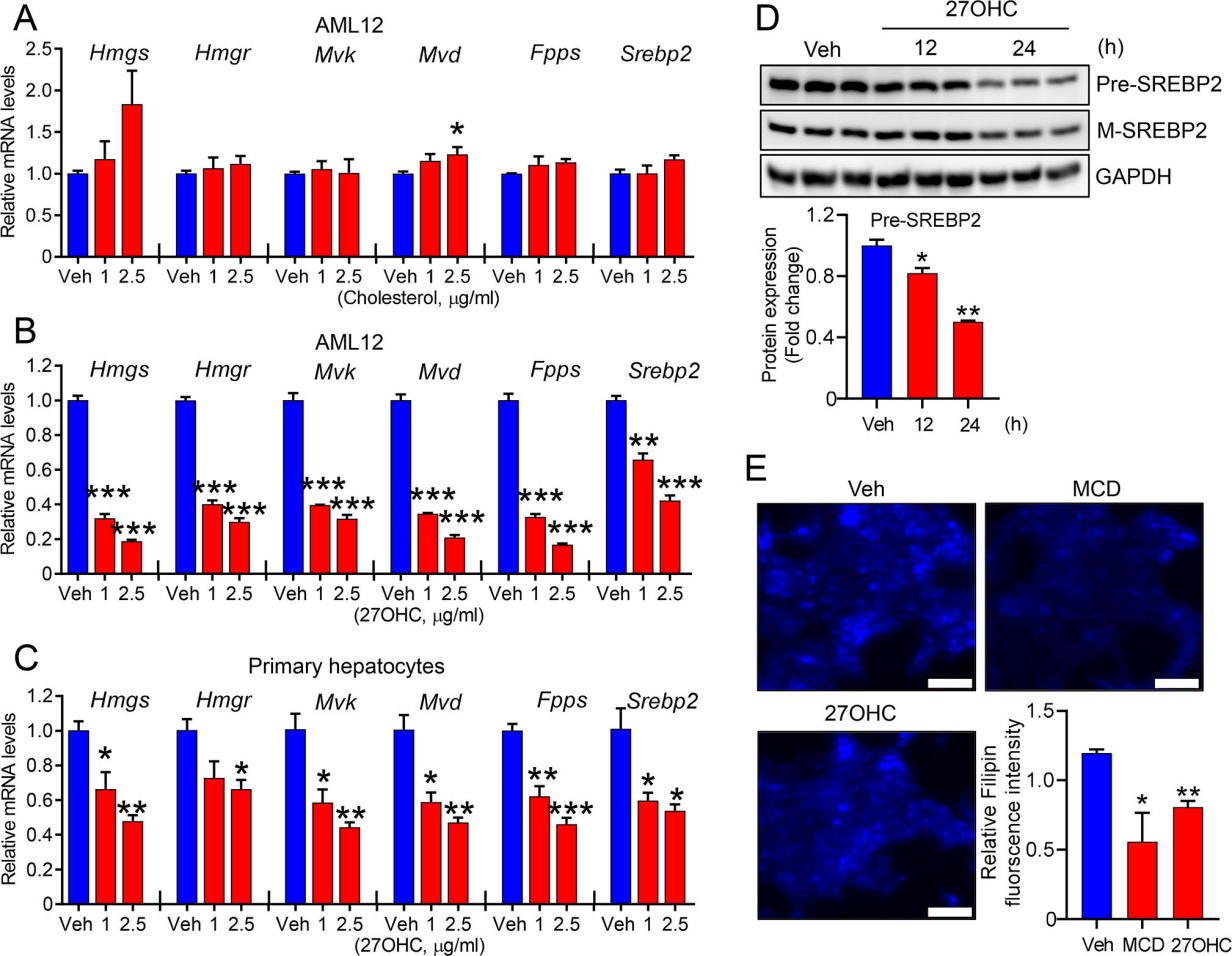
27-Hydroxycholesterol, but not cholesterol, inhibits the expression of genes related to the mevalonate pathway. AML12 cells were incubated with the indicated concentrations of cholesterol (**A**) or 27OHC (**B**) for 24 h. The expression of *Hmgs, Hmgr, Mvk, Mvd, Idi1, Fpps,* and *Srebp2* genes was measured using qPCR. The expression of each gene was normalized to that of *Gapdh*. (**C**) Mouse primary hepatocytes were incubated with 27OHC or the vehicle control for 24 h. The expression of indicated genes was measured using qPCR. (**D**) Following 12 h or 24 h incubation with 2.5 μg/ml 27OHC, total lysates were prepared from AML12 cells to detect precursor and mature SREBP2 proteins using immunoblot assay. (**E**) Total cholesterol levels in AML12 cells were quantified using filipin staining after incubation with 5 mM methyl-β-cyclodextrin (MCD) or 2.5 μg/ml 27OHC (scale bars, 50 μm). *n*=3 independent biological replicates. Values are presented as the mean ± S.E.M. *P<0.05, **P<0.01, and ***P<0.001.

To validate the identified enriched proteins and pathway underlying the suppression of cholesterol synthesis following 27OHC treatment, the expression of genes related to the MVA pathway in hepatocytes was evaluated using qRT-PCR. Dose–response experiments indicated that no significant differences were observed in the mRNA levels of the genes related to the MVA pathway, such as HMG-CoA synthase (*Hmgs*), HMG-CoA reductase (*Hmgr*), mevalonate kinase (*Mvk*), mevalonate diphosphate decarboxylase (*Mvd*), farnesyl pyrophosphate synthase (*Fpps*), and sterol regulatory element-binding protein 2 (*Srebp2*), up to 2.5 μg/ml of cholesterol (Figure 3A). In contrast, a significant reduction in the mRNA levels of the genes related to the MVA pathway was observed in 27OHC-treated AML12 cells (Figure 3B) and primary hepatocytes (Figure 3C). SREBP2 is a master transcription factor that increases cholesterol biosynthesis when cholesterol levels are depleted. To determine whether 27OHC also affected SREBP2 protein levels, we treated the cells with 27OHC for the indicated time (Figure 3D). As a result, SREBP2 expressions were clearly abolished when the cells were treated with 27OHC (Figure 3D). To quantify the intracellular cholesterol levels, cells were stained with filipin dye following 27OHC treatment for 24 h. As previously reported [33], the cholesterol-depleting agent methyl-β-cyclodextrin demonstrated reduced filipin staining. Similarly, the fluorescence intensity indicated a significant reduction in filipin fluorescence in 27OHC-treated cells (Figure 3E).

### 27OHC inhibits glycolysis and extracellular acidification rate

**Figure 4:**
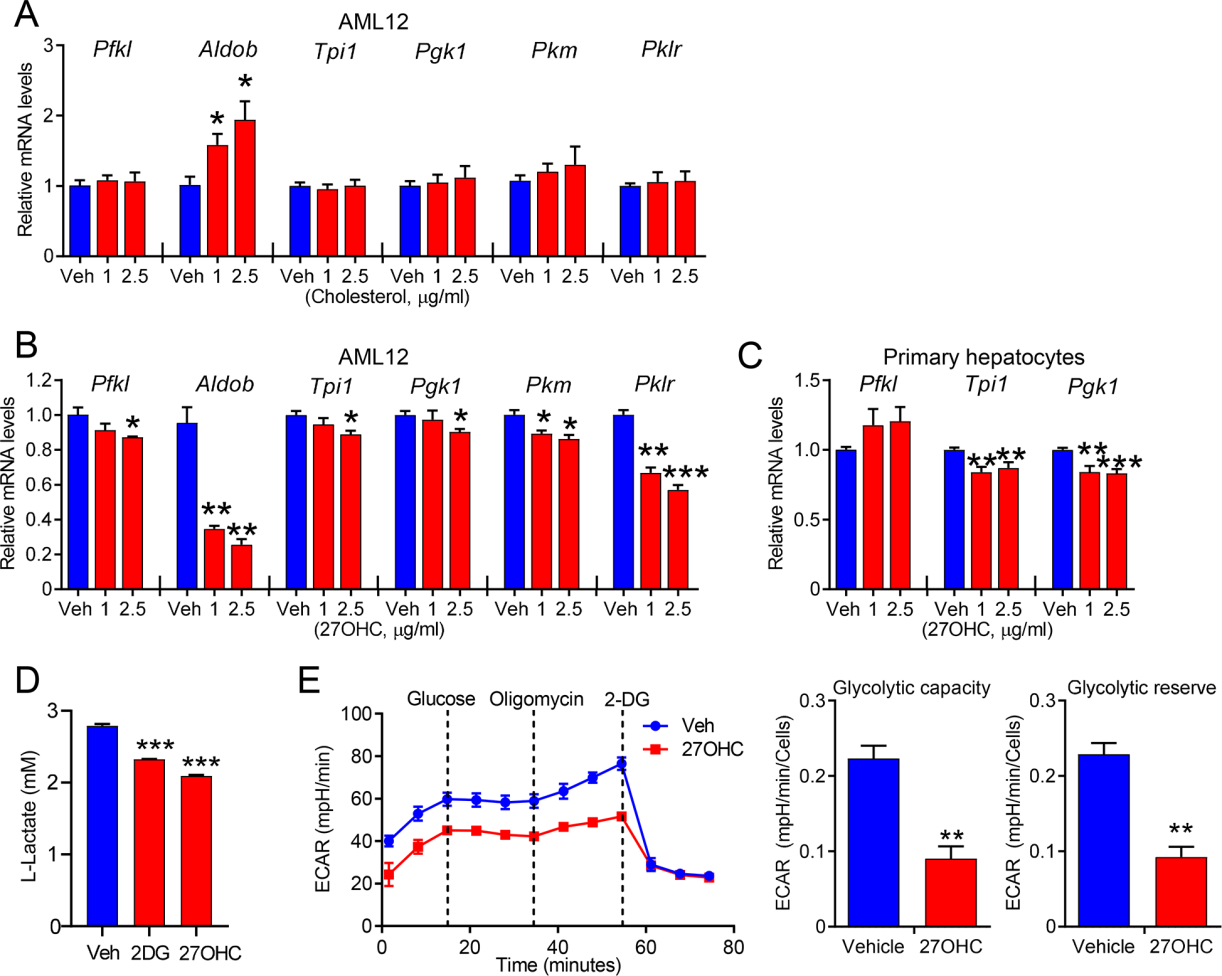
The glycolytic pathway is significantly suppressed by 27-hydroxycholesterol. AML12 cells were incubated with different doses of cholesterol (**A**) or 27OHC (**B**) for 24 h. The expression of *Pfkl, Aldob, Tpi1, Pgk1, Pkm,* and *Pklr* genes was measured using qPCR. (**C**) Mouse primary hepatocytes were incubated with 27OHC or vehicle control for 24 h. The expression of the *Pfkl, Tpi1,* and *Pgk1* genes was measured using qPCR. (**D**) Inhibition of l-lactate production following treatment with 27OHC (2.5 μg/ml) for 48 h was assessed using the glycolysis assay kit. 2-DG was used as the positive control. (**E**) Serial measurements of the extracellular acidification rate (ECAR, left). Glycolytic capacity and glycolytic reserve of cell (right) were quantified following 27OHC treatment (2.5 μg/ml). *n*=3 independent biological replicates. Values are presented as the mean ± S.E.M. *P<0.05, **P<0.01, and ***P<0.001.

Next, we examined whether 27OHC affected the expression of the glycolysis-related genes, as proteomic analysis indicated that glycolysis was negatively modulated by 27OHC (Figure 2D). Similar to the genes related to the MVA pathway, no difference was observed in the expression of key glycolytic genes between the vehicle and cholesterol treatments (Figure 4A). Meanwhile, 27OHC significantly reduced the expression of glycolytic genes, such as 6-phosphofructokinase (*Pfkl*), aldolase B (*Aldob*), triosephosphate isomerase 1 (*Tpi1*), phosphoglycerate kinase 1 (*Pgk1*), and pyruvate kinase (*Pklr*), in AML12 cells (Figure 4B). However, no notable effects were observed on the expression of gluconeogenic genes (Supplementary Figure 2A). The expression of only *Tpi1* and *pgk1* genes was significantly suppressed in primary hepatocytes (Figure 4C). Consequently, we hypothesized that this down-regulation would lead to reduced glycolytic activity, as these enzymes are crucial for glycolysis. As shown in Figure 4d, the glycolysis inhibitor, 2-deoxy-d-glucose, reduced lactate production, and this inhibition was much more pronounced following 27OHC treatment (Figure 4D). Finally, extracellular acidification rate (ECAR), which reflects glycolytic activity, was analyzed following 27OHC treatment using a glycolysis stress test. Under basal conditions, the ECAR significantly decreased (60%) in cells treated with 27OHC. Moreover, after adding glucose and oligomycin, which stimulate glycolysis to its maximum capacity, 27OHC-treated cells demonstrated a significantly lower maximal ECAR than the control cells (Figure 4E). Therefore, 27OHC limits the glycolytic capacity both at baseline and under stress conditions. Meanwhile, 27OHC did not affect overall mitochondrial abundance, as quantification of the mtDNA to nuclear DNA ratio and mitochondrial gene expression revealed no significant differences between vehicle- and 27OHC-treated cells (Supplementary Figure 2B and C).

### 27OHC reduces ROS and HIF-1α levels in hepatocytes

**Figure 5:**
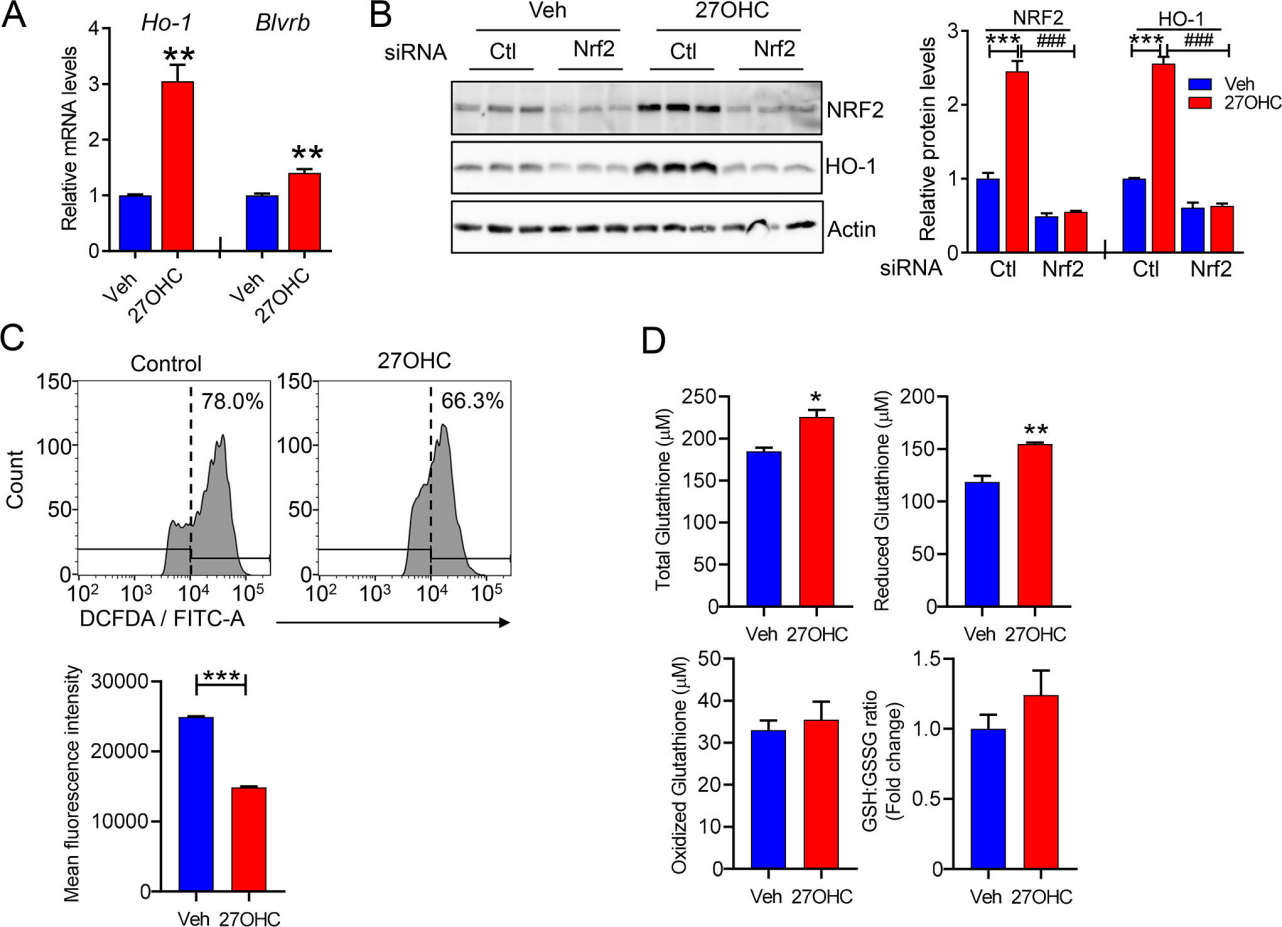
Effects of 27-hydroxycholesterol on ROS generation and antioxidant responses in hepatocytes. (**A**) Relative mRNA levels of *Ho1* in AML12 cells treated with either vehicle or 2.5 µg/ml 27OHC for 24 h. (**B**) NRF2 and HO-1 protein levels following 27OHC (2.5 μg/ml) treatment of Nrf2 siRNA-transfected AML12 cells were assessed using immunoblotting. Actin was used as the loading control. (**C**) Flow cytometry analysis of reactive oxygen species (ROS) levels in AML12 cells treated with vehicle or 2.5 µg/ml 27OHC for 24 h. ROS levels were detected using DCFDA/FITC-A staining. Mean fluorescence intensity (MFI) indicates the proportion of cells with elevated ROS levels. (**D**) Total glutathione (top left), reduced glutathione (GSH, top right), oxidized glutathione (GSSG, bottom left), and GSH:GSSG ratio (bottom right) were determined in AML12 cells treated with either vehicle or 2.5 µg/ml 27ohc for 24 h. *n*=3 independent biological replicates. Values are presented as the mean ± S.E.M. **P<0.01.

Previous studies have shown that ROS play a significant role in regulating cholesterol synthesis and glycolysis [34,35]. We measured the mRNA levels of heme oxygenase-1 (*Ho1*) and biliverdin reductase b (*Blvrb*), which are important antioxidant enzymes that play critical roles in cellular defense mechanisms against oxidative stress by catalyzing heme protein degradation, to understand the mechanistic details of how 27OHC influences cholesterol and glucose metabolism. A considerable induction of *Ho1* transcripts was observed following 27OHC treatment (Figure 5A), which further supported the functional annotation of the increased heme degradation pathway using the proteomic analysis (Figure 2F). NRF2, a transcription factor that regulates HO-1 expression, was also increased following 27OHC, whereas HO-1 protein expression was significantly attenuated when the cells were transfected with Nrf2 siRNA (Figure 5B). As expected, the cellular ROS levels, as measured based on the fluorescence intensity of 2,7-dichlorofluoroscin diacetate (DCFDA), were significantly decreased following 27OHC treatment (Figure 5C). In line with decreased ROS levels, total glutathione concentrations and reduced glutathione (GSH) levels were significantly increased following 27OHC treatment. In contrast, oxidized glutathione (GSSG) levels showed no significant difference between the 27OHC and vehicle groups, indicating that the increase in total glutathione was primarily related to elevated GSH levels (Figure 5D). Therefore, 27OHC effectively inhibited ROS generation and enhanced the antioxidant capacity of hepatocytes.

**Figure 6:**
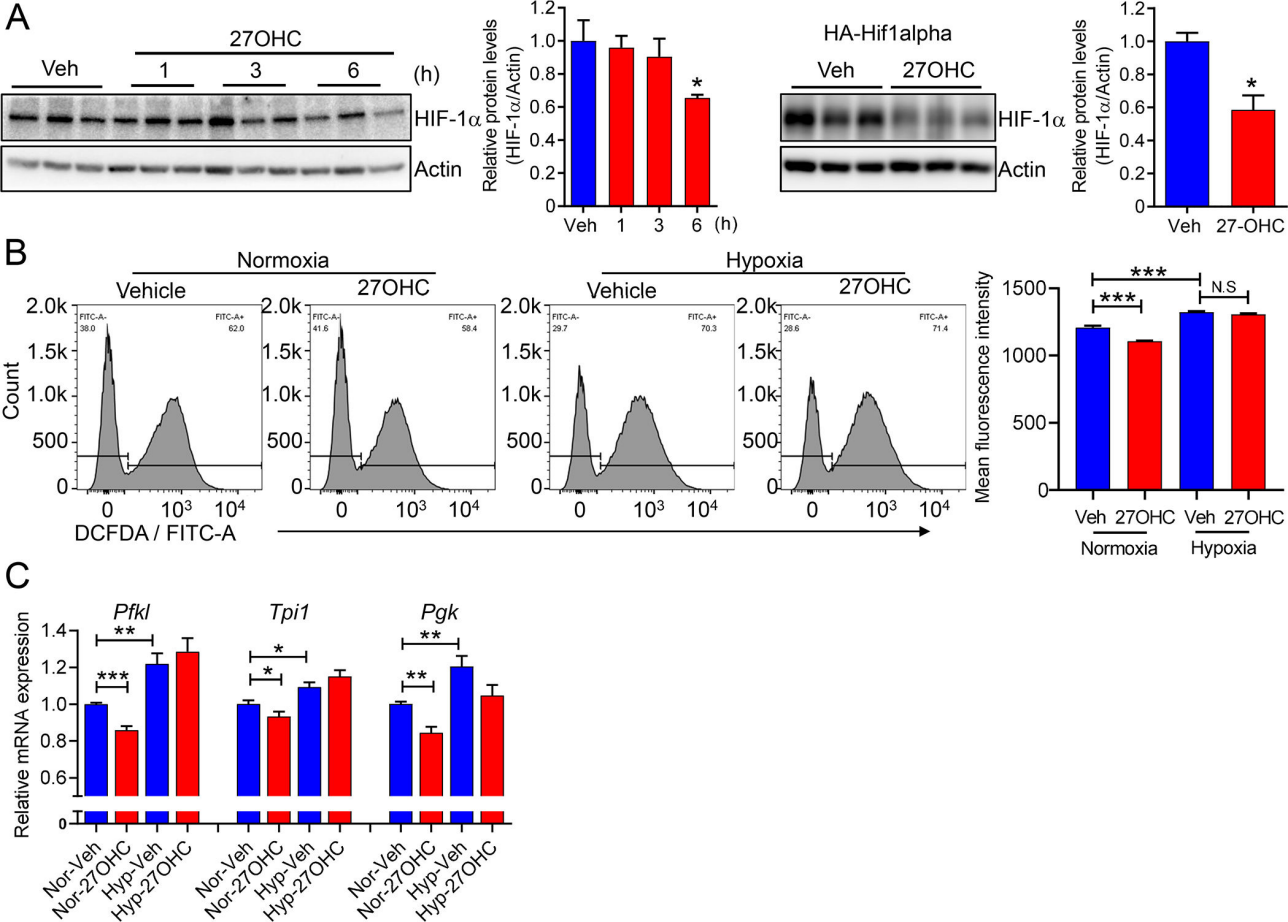
Inhibition of HIF-1α following 27-hydroxycholesterol treatment contributes to decreased glycolytic gene expression. (**A**) Expression levels of endogenous (left) and transfected HIF-1α (right) were determined in whole-cell lysates following 27OHC (2.5 μg/ml) treatment. Quantitative analysis of HIF-1α expression was presented in a bar graph. β-Actin was used as the loading control. (**B and C**) AML12 cells were grown under normoxic or hypoxic (2% O_2_) conditions for 24 h with 27OHC. (**B**) Intracellular ROS levels were detected using DCFDA/FITC-A staining. (**C**) Glycolytic gene expression under hypoxic conditions was analyzed. *n*=3 independent biological replicates. Values are presented as the mean ± S.E.M. *P<0.05, **P<0.01, and ***P<0.001.

Under normoxic conditions, HIF-1α is hydroxylated by prolyl hydroxylase domain (PHD) enzymes, leading to its ubiquitination and degradation by proteasomes. As ROS inhibits enzyme activity of the PHD [36], we further evaluated whether ROS suppression following 27OHC treatment would result in lower HIF-1α levels. Notably, western blot results demonstrated that 27OHC treatment significantly decreased HIF-1α protein levels in both endogenous and overexpressed conditions (Figure 6A), and the addition of 1 μM MG132 to these conditions prevented the suppression of HIF-1α (Supplementary Figure 3A), demonstrating 27OHC increased proteasome-mediated HIF-1α degradation. As expected, the inhibition of HIF-1α by 27OHC was largely reversed following treatment with high levels of ROS in a hypoxic chamber (Figure 6B, Supplementary Figure 3B ) [37]. Concurrently, 27OHC no longer suppressed the expression of glycolytic genes under HIF-1α stabilization (Figure 6C). Meanwhile, the mRNA levels of the MVA pathway-related genes were not affected by hypoxia (data not shown). Therefore, 27OHC affected glycolysis via the ROS–HIF-1α axis.

### 27OHC attenuates lipid accumulation in an *in vitro* MASH model

**Figure 7:**
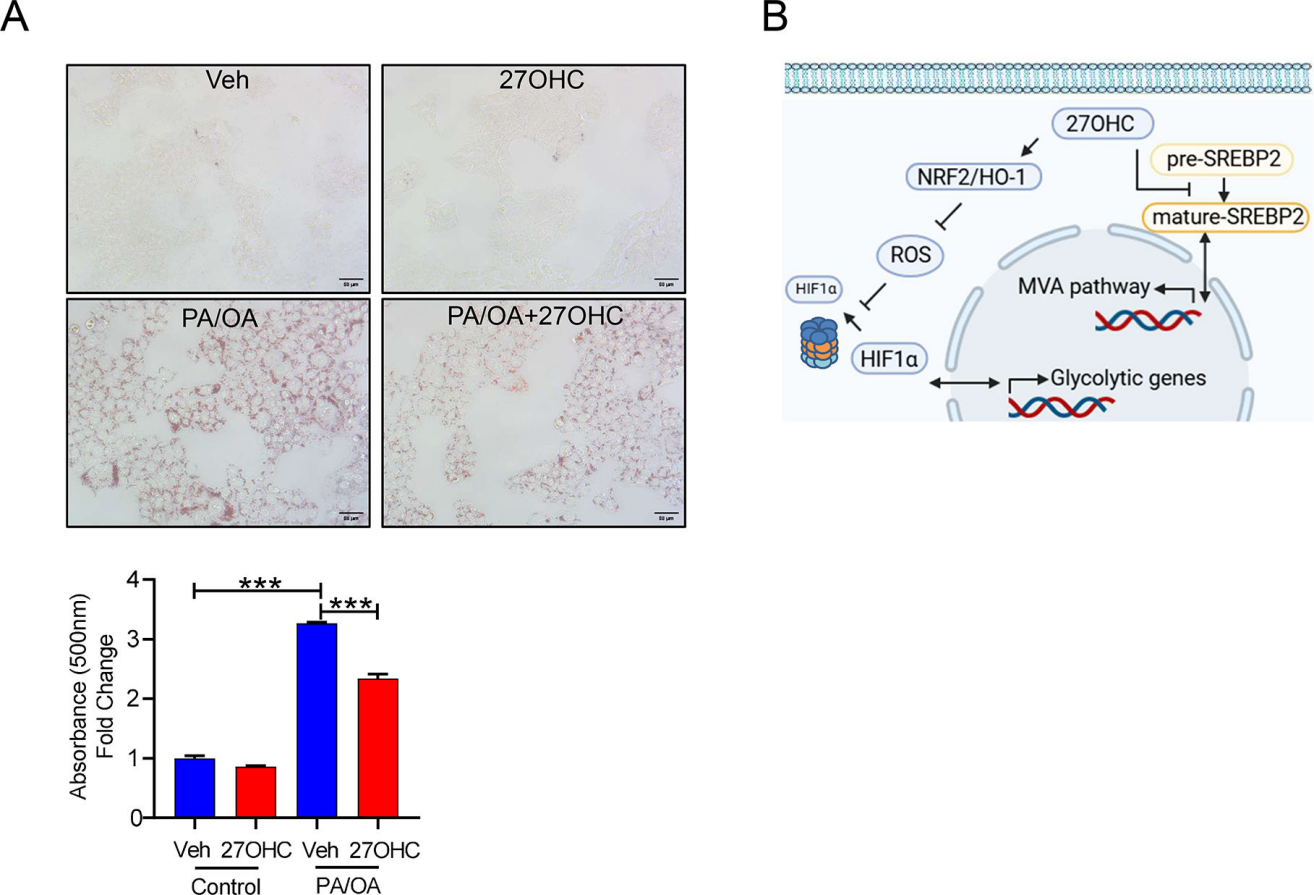
27-Hydroxycholesterol inhibits lipid accumulation in hepatocytes. (**A**) Oil Red O staining was used to evaluate intracellular lipid accumulation in cells treated with vehicle, 27OHC, palmitic/oleic acid (PA/OA), or PA/OA in combination with 27OHC (2.5 μg/ml). Scale bars: 50 µm. Lower panel indicated the quantification of Oil Red O absorbance at 500 nm. Data are presented as fold change relative to vehicle control. *n*=3 independent biological replicates. Values are presented as the mean ± S.E.M. ***P<0.001. (**B**) Working model.

To assess the effect of 27OHC on an *in vitro* metabolic dysfunction-associated steatohepatitis (MASH) model system, Oil Red O staining was performed on cells treated with saturated and monounsaturated fatty acids. Representative microscopic images (left panel) revealed minimal lipid accumulation in both vehicle- and 27OHC-treated cells, whereas palmitic/oleic acid (PA/OA) treatment induced prominent lipid droplet formation (Figure 7A). Importantly, co-treatment with PA/OA and 27OHC resulted in a noticeable reduction in lipid accumulation compared with PA/OA alone. Quantification of Oil Red O absorbance at 500 nm (lower panel) confirmed these observations. These data suggest that 27OHC efficiently attenuates fatty acid-induced lipid accumulation in hepatocytes.

## Discussion

In this study, we demonstrated that 27OHC inhibits both the MVA and glycolytic pathways in hepatocytes. To the best of our knowledge, this is the first study to provide information on metabolic alterations caused by 27OHC in hepatocytes using proteomic analysis. The activation of the NRF2/HO-1 pathway, which plays a critical role in the cellular defense against oxidative stress, can contribute to these metabolic changes by modulating the cellular redox status and inhibiting pathways sensitive to oxidative stress, such as glycolysis. As these pathways are crucial for maintaining the lipid balance, their inhibition may represent a molecular mechanism by which 27OHC suppresses lipid accumulation in the liver (Figure 7B).

Oxysterols can be formed enzymatically by the action of some cytochrome P450 family members or non-enzymatically by the action of ROS. Notably, some oxysterols inhibit cholesterol synthesis. In particular, 25OHC inhibits HMG-CoA reductase, a key enzyme in the MVA pathway responsible for cholesterol synthesis [38]. By inhibiting this enzyme, 25OHC effectively reduces the overall biosynthesis of cholesterol in the body and influences the expression of genes involved in lipid metabolism. In addition, 24SOHC, a product of CYP46A1, decreases the expression of enzymes involved in cholesterol and isoprenoid synthesis in neurons via SREBP-2 modification [39]. As 24SOHC, 25OHC, and 27OHC belong to the same class of oxysterols generated through enzymatic hydroxylation reactions by the cytochrome P450 family members, these oxysterols likely share a common feature in terms of cholesterol regulation. Interestingly, high intracellular cholesterol levels of approximately 10–25 µg/ml affect the MVA pathway [40,41]. However, our results revealed that 1 µg/ml of 27OHC sufficiently inhibited cholesterol synthesis. Therefore, 27OHC is significantly more effective than cholesterol in inhibiting cholesterol synthesis. Previous studies have shown that AMPK inhibits cholesterol synthesis by suppressing the phosphorylation of the key enzyme 3-hydroxy-3-methylglutaryl-CoA reductase (HMGCR) [42]. However, our knockdown experiments have indicated that AMPK was not involved in the 27OHC-induced suppression of the MVA pathway (Supplementary Figure S4A, B). Li et al. reported that 27OHC inhibits the cleavage and activation of SREBP-1c, which is involved in fatty acid synthesis, by promoting the interactions between Insig-2 and SREBP-1 in the endoplasmic reticulum (ER) membrane [13]. As SREBP-2 activity is tightly regulated by interactions with SREBP cleavage-activating protein (SCAP) and Insig proteins, we speculated that 27OHC could also induce the retention of the SCAP/SREBP-2 complex in the endoplasmic reticulum to inhibit SREBP-2 activation.

The mechanisms through which 27OHC increased the hepatic NRF2 levels in the present study are not yet clear. Under normal conditions, NRF2 is bound to its inhibitor, Keap1, which facilitates its degradation by the proteasome. Oxidative stress typically modifies cysteine residues on Keap1, inducing a conformational change that prevents Keap1 from tagging NRF2 for degradation. Vurusaner et al. reported that 27OHC induces a pro-survival response in human promonocytic cells by increasing NRF2 [43] and suggested that 27OHC (10 μM) induces NRF2 activation by causing an early but temporary increase in cellular ROS levels, which in turn enhances the phosphorylation of the MEK–ERK and PI3K–Akt pathways. However, our results indicated that 27OHC largely suppressed cellular ROS levels at an early time point, which was comparable to the 24-h treatment (Supplementary Figure S4). Furthermore, neither AKT nor ERK was activated by 27OHC in hepatocytes (Supplementary Figure S4). Therefore, we hypothesized that different molecular mechanisms may exist for 27OHC-induced NRF2 activation, depending on the cellular context. Liver X receptors and estrogen receptors (ERs) have been identified as 27OHC receptors in various tissues [10]. Notably, estrogen receptors exert antioxidant effects partly by activating NRF2 [44,45]. Based on these studies, estrogen increases the expression of NRF2 and its target antioxidant genes, thereby contributing to cellular protection against oxidative stress. We anticipate evaluating the involvement of estrogen receptors in NRF2 activation and ROS suppression by 27OHC in hepatocytes in future studies.

By analyzing proteomes and glycolytic flux, we demonstrated that 27OHC largely suppressed the glycolysis pathway. Notably, many components of glycolysis, including HIF-1α and pyruvate kinase M, are up-regulated in the hepatocytes of fatty liver, and increased glycolysis in metabolic dysfunction-associated steatohepatitis is a significant metabolic alteration that contributes to its progression [46]. Therefore, 27OHC-mediated inhibition of glycolysis represents a promising therapeutic strategy for treating fatty liver disease, as it can reduce the supply of glycolytic intermediates that feed into lipogenesis. Although reduced ROS and HIF-1α levels partially contribute to glycolysis inhibition, membrane instability can also be a possible explanation for the altered metabolism. Notably, a recent study showed that 27OHC impairs prostate cancer cell signaling by disrupting lipid rafts and inhibiting the IL6–JAK–STAT3 pathway [47]. Considering that STAT3 directly regulates the expression of several glycolytic genes and that physical interaction between STAT3 and HIF-1α mediates the physiological response [48,49], it is very likely that 27OHC may also influence the cellular membrane composition by lowering cholesterol metabolism, which, in turn, can suppress intracellular signaling pathways, which are responsible for transcriptional activation of glycolysis. Finally, as 27OHC also exerts antitumor effects in certain cancers and cancer cells often rely on glycolysis for energy production (a phenomenon known as the Warburg effect), exploring an association between the antitumor activity of 27OHC and glycolysis inhibition is crucial.

In conclusion, this study provides compelling evidence that 27OHC significantly inhibits cholesterol synthesis, glycolysis, and ROS generation in hepatocytes. In line with previous studies that suggested the inhibition of lipid accumulation and hepatic inflammation following 27OHC treatment [13,14], our data revealed additional mechanisms through which 27OHC prevents MAFLD. Nevertheless, future research should evaluate the long-term effects of 27OHC in various diet-induced animal models of MAFLD and assess the metabolic changes in distinct tissues, including the liver, for clinical applications.

## Materials and methods

### Cell culture and material treatment

AML12 cells were cultured in Dulbecco’s modified Eagle medium/F12 (Hyclone; cat. no. SH30023.01) supplemented with 10% fetal bovine serum (Gibco; cat. no. 16000–044), 1% penicillin/streptomycin (Gibco; cat. no. 15140–122), and 40 ng/mL dexamethasone in a 5% CO_2_ humidified chamber at 37°C. 27OHC was purchased from Tocris Bioscience (cat. no. 3907). HA-HIF1alpha-pcDNA3 [50] was a gift from William Kaelin (Addgene plasmid # 18949)

### Primary hepatocyte isolation

Eight-week-old C57BL/6 male mice were purchased from Hana Bio Company (Pusan, Korea). Mice were housed in a temperature-controlled 12:12 h light-dark cycle facility with free access to water and normal chow diet. Primary hepatocytes were isolated from adult male C57BL/6 mice using a two-step collagenase perfusion method as previously described [51]. Briefly, the mice were anesthetized under 1.5% isoflurane vaporized by oxygen at a flow rate of 1.0 l/min. Once the mice became nonreactive to painful stimuli after inhalation of anesthesia gas, cervical dislocation was used for euthanasia. Subsequently, the mouse abdomen surface was sanitized with 70% ethanol, and the peritoneal cavity was opened using surgical scissors. The liver was perfused with liver perfusion medium (Gibco; cat. no. 17701038) *in situ* via the portal vein for 10 min at 37°C for 20 min, followed by Dulbecco's Modified Eagle Medium (DMEM)-low glucose with 1% penicillin/streptomycin and 15 mM HEPES containing type IV collagenase. The liver was then removed and gently minced, and the released cells were dispersed in DMEM (high glucose) containing serum-free 1% penicillin/streptomycin, 15 mM HEPES, and 0.1 µM dexamethasone. The solution containing the mixed cells and debris was passed through a 70-µm cell strainer. Hepatocytes were inoculated into collagen-coated plates (6 × 10^5^ cells/well in 6-well plates) in Medium 199 (Sigma-Aldrich; cat. no. M4530) with 10% fetal bovine serum, 1% penicillin/streptomycin, 5 mM HEPES, and 0.01 μM dexamethasone. All animal experiments were performed in accordance with the National Institutes of Health Guide for the Care and Use of Laboratory Animals, and the protocols were approved by the Pusan National University Institutional Animal Care and Use Committee (PNU-IACUC; approval no. PNU-2024–0282).

### Cell viability assays

For the WST1 assay, AML12 cells were seeded in 96-well culture plates at a density of 5 × 10^4^ cells/well and cultured overnight. The cells were then treated with various concentrations of 27OHC for 24 h. Next, 10 µl of WST1 reagent (Sigma-Aldrich; cat. no. 5015944001) was added to each well, and the cells were incubated for 1 h in a CO_2_ incubator at 37°C. The absorbance of the assay solution was measured at 450 nm with a reference wavelength of 620 nm. The assay was performed following the manufacturer’s instructions.

### TUNEL assay

The TUNEL assay was performed using a one-step TUNEL *in situ* apoptosis kit (Elabscience Biotechnology; cat. no. E-CK-A-320) to detect apoptotic AML12 cells following the manufacturer’s instructions. Cells were fixed with 4% paraformaldehyde (Biosesang; cat. no. FR2013-100-00) for 20 min at room temperature. Permeabilization was performed using 0.2% Triton X-100 (Biosesang; cat. no. TR1020-500-00) for 10 min at 37°C. The cells were then treated with DNase I working solution (positive control) or DNase I buffer for 30 min at 37°C and incubated with TdT buffer for 30 min at 37°C, followed by staining with a labeling working solution for 1 h at 37°C. Subsequently, the cells were treated with DAPI for 5 min at room temperature. The broken DNA was labeled with fluorescein isothiocyanate (FITC), and the nuclei were stained with DAPI. Imaging was performed using a fluorescence microscope.

### Peptide preparation and liquid chromatography–tandem mass spectrometry (LC–MS/MS) analysis

AML12 cells were treated with 27OHC for 24 h. After washing with phosphate-buffered saline (PBS), cells were harvested via centrifugation (4000 ***g***, 5 min). For in-solution digestion, the cells were dissolved in 0.2% ProteaseMAX (Promega, Madison, WI, U.S.A.; cat. no. V2071) in 40 mM ammonium bicarbonate (ABC, NH_4_HCO_3_). After sonication at 30% amplitude for 3 s for ten times, the cell lysates were four-fold diluted with 40 mM ABC. The cells were then incubated with 10 mM dithiothreitol at 56°C for 20 min, followed by incubation with 20 mM iodoacetamide at room temperature in the dark for 20 min. The proteins were then estimated using the BCA assay, and a trypsin Lys-C (1:50) mixture (V5073; Promega) was added to 100 µg of protein and incubated overnight at 37°C. After centrifugation at 16,000×g for 10 s at 4°C, the supernatant was collected in a new E-tube. The peptides were treated with 0.5% trifluoroacetic acid (Thermo Fisher Scientific, San Jose, CA, U.S.A.; cat. no. 28901) for 5 min at 25°C to stop the reaction. After drying, the peptides were applied to a C18 spin column (Thermo Fisher Scientific; cat. no. 89870), and the eluents were dried using a lyophilizer.

For LC–MS/MS analysis, tryptic-digested peptides were analyzed using a Q-Exactive Plus hybrid quadrupole orbit trap mass spectrometer (Thermo Fisher Scientific) interfaced with an EASY-Spray source. Chromatographic separation of the injected peptides was achieved using an Ultimate 3000 RSLCnano System (Thermo Fisher Scientific) equipped with an Acclaim PepMap^TM^ 100 (75 µm×2 cm, 3 µm, NanoViper, Thermo Fisher Scientific) loading column and an EASY-Spray column PepMap^TM^ RSLC C18 (75 µm×50 cm, 2 µm, Thermo Fisher Scientific) separation column. The tryptic-digested peptides were loaded using an RS autosampler and separated using a linear gradient of ACN/water containing 0.1% formic acid at a flow rate of 300 nl/min. The LC eluent was electrosprayed from an analytical column, and a voltage of 2.0 kV was applied via the liquid junction of the nanospray source. Peptide mixtures were separated using a 5–40% ACN gradient for 40 min. The analysis method involved a complete MS scan with a range of 350–2000 m/z and data-dependent MS/MS (MS2) of the ten most intense ions detected using the complete MS scan. The mass spectrometer was programmed to acquire data in data-dependent acquisition mode. The mass spectrometer was calibrated using the proposed calibration solution following the manufacturer’s instructions. Tandem mass spectra were processed using the software Proteome Discoverer version 2.4 SP1 (Thermo Fisher Scientific) to perform a database search. The spectral data were searched against the mouse UniProt database (release version 2020_06). The analysis workflow included four nodes: Spectrum Files (data input), Spectrum Selector (spectrum and feature retrieval), Sequest HT (sequence database search), and Percolator (peptide spectral match or PSM validation and FDR analysis). All identified proteins had an FDR of ≤1%, which was calculated at the peptide level and validated based on the q-value. Search parameters allowed for tryptic specificity of up to two missed cleavages, with carbamidomethylation of cysteine as a fixed modification and oxidation of methionine as a variable modification. The mass search parameters for +1, +2, and +3 ions included mass error tolerances of 20 ppm for precursor ions and 0.5 Da for fragment ions. After searching the database, the label-free quantitative analysis was performed. A normalized peptide spectrum match index was used to calculate the quantitative changes in the identified proteins between the experimental groups. The peptide spectrum match index was calculated for each protein containing a cumulative peptide spectrum match from each technical replicate. The G‐test was performed for peptide spectrum matches to estimate the statistical confidence for fold changes in identified proteins between the experimental groups [52–56].

### Bioinformatics

IPA was used for in-depth bioinformatic analysis. For the identified proteins, UniProt protein accession numbers coupled with the values of normalized fold changes from proteomics were uploaded to IPA based on the protein expression criteria. We used the following criteria for quantitative pathway analysis: Z-score cutoff = 2.0, –log (*P*-value) > 2.5.

### FACS assay and glutathione quantification

Flow cytometry data were acquired using a BD FACSCanto 2 flow cytometer and analyzed using FlowJo software (Tree Star, Inc., U.S.A.). AML12 cells were collected in FACS buffer comprising 2% FBS in PBS and incubated with FITC–Annexin V (BD Biosciences; cat. no. 556419) with Annexin V binding buffer for 30 min at room temperature in the dark, and PerCP-7AAD (BD Biosciences; cat. no. 559925) was then added immediately before analysis. 5-Fluorouracil (Sigma-Aldrich; cat. no. F6627-1G) was used as the positive control. Intracellular ROS levels were measured by staining the cells with DCFDA (Sigma-Aldrich; cat. no. 287810). Total glutathione, GSH, GSSG, and GSH:GSSG ratio levels were measured using the GSH+GSSG/GSH colorimetric assay kit (Colorimetric) (ab239709, Abcam, Cambridge, U.K) following the manufacturer’s instructions. The absorbance was measured at 405 nm using a microplate reader (Synergy H1, Biotek, U.S.A.).

### RNAi experiment

AML12 cells were transfected with Nrf2 small-interfering RNA (Santa Cruz Biotechnology, Inc.; cat. no. sc-37049) or negative control siRNA (Bioneer, Inc.; cat. no. SN-1002) using Lipofectamine 2000 (Invitrogen, Carlsbad, CA, U.S.A.), following the manufacturer’s protocol. Nrf2 siRNA (sc-37049) is a pool of three target-specific 19–25 nt siRNAs designed to knock down gene expression.

### Immunoblotting

The treated cells were collected, and the total protein was isolated for immunoblotting. AML12 cells were lysed in cold lysis buffer (10 mM pyrophosphate, 10 mM glycerophosphate, 50 mM NaF, 1.5 mM Na3VO4, 40 mM HEPES, pH 7.5, 120 mM NaCl, 1 mM PMSF, 5 mM MgCl_2_, 0.5% Triton X-100, and protease inhibitor cocktail). The lysates were briefly sonicated, denatured through heating at 99°C for 2 min and then subjected to SDS–PAGE (10% acrylamide gel). The proteins were then electrophoretically transferred onto nitrocellulose membranes. The membranes were blocked with 5% nonfat milk in Tris-buffered saline containing 0.05% Tween 20 (TBS-T) and incubated overnight at 4°C with the following primary antibodies: HO-1 (E3F4S) rabbit mAb (Cell Signaling Technology; cat. no. 43966), NRF2 (D1Z9C) XP® rabbit mAb (Cell Signaling Technology; cat. no. 12721), HIF-1α (D2U3T) rabbit mAb (Cell Signaling Technology; cat. no. 14179), AMPKα (Cell Signaling Technology; cat. no. 2532), phospho-Akt Ser473 (Cell Signaling Technology; cat. no. 9271), Akt (Santa Cruz Biotechnology, Inc.; cat. no. sc-5298), SREBP2 (Invitrogen, Inc.; cat. no. PA1-338), and β-actin (Cell Signaling Technology; cat. no. 4967). The membranes were then washed thrice with TBS-T, incubated with horseradish peroxidase-conjugated secondary antibodies, and washed six times with TBS-T, and protein bands were detected using an enhanced chemiluminescence system (GE Healthcare, Chicago, U.S.A.). Densitometric analysis was performed using the Amersham Imager 680 Analysis Software.

### Enzymatic lactate measurements

AML12 cells (1 × 10^5^ cells/well in 6-well plates) were treated with 27OHC for 48 h, and the cell culture medium was collected. l-Lactate production in the medium was measured using an EnzyChrom Glycolysis Assay Kit (BioAssay Systems; cat. no. ECGL-100) following the manufacturer’s protocol.

### Seahorse XF assays

The ECAR, an indicator of mitochondrial glycolytic activity, was measured using the Mito Stress Test on an Agilent Seahorse XFp Analyzer (Agilent Technologies Inc., California, U.S.A.) following the manufacturer’s instructions. AML12 cells were pretreated with 27OHC for 48 h in eight-well plates. Prior to the assay, the medium was replaced with Seahorse XF DMEM (Agilent Technologies Inc.; cat. no. 103575–100) containing 2 mM glutamine. The cells were incubated in a non-CO_2_ incubator at 37°C for 1 h. Then, 10 mM glucose, 10 µM oligomycin, and 50 mM 2-deoxyglucose–glucose (2-DG) solutions were consecutively added to each well to assess the glycolytic parameters, including the glycolysis rate following glucose addition, maximum glycolytic capacity with oligomycin, and glycolytic involvement using 2-DG. Data were normalized to cell numbers and analyzed using Wave software (Agilent Technologies Inc.).

### Oil Red O staining

AML12 cells were seeded in six-well plates and cultured under standard conditions (37°C, 5% CO₂). To mimic MASH condition *in vitro*, cells were treated with BSA-conjugated palmitic acid (Sigma-Aldrich; cat. no. P9767)/oleic acid (Sigma-Aldrich; cat. no. O7501) with a molar ratio of 1:2, resulting in final concentrations of 250 µM PA and 500 µM OA, as previously described [57]. After treatment, cells were washed twice with PBS and fixed with 4% paraformaldehyde for 60 min at room temperature. Oil Red O stock solution (0.5% w/v in isopropanol) was diluted 3:2 with distilled water to prepare the working solution, which was then filtered. Fixed cells were stained with the working solution for 15–30 min at room temperature. Excess stain was removed by rinsing with 60% isopropanol, followed by washes with distilled water. Lipid accumulation was visualized under a light microscope. For quantitative analysis, Oil Red O was eluted with 100% isopropanol, and the absorbance was measured at 500 nm using a microplate reader.

### Quantitative RT-PCR

Total RNA was extracted using an Easy-BLUE Total RNA Extraction Kit (iNtRON Biotechnology; cat. no. 17061) and used to synthesize cDNA using a High-Capacity cDNA Archive Kit (Applied Biosystems; cat. no. 4368813) following the manufacturer’s instructions. Real-time PCR was performed using SYBR Green PCR Master Mix (Applied Biosystems; cat. no. 4309155) and ABI QuantStudio 3 (Applied Biosystems, Inc., U.S.A.). The oligonucleotide sequences used in this study are listed in Table 2.

**Table 2:** Primer sequence used for quantitative PCR.

Gene	Forward	Reverse
*Hmgs*	GCCGTGAACTGGGTCGAA	GCATATATAGCAATGTCTCCTGCAA
*Hmgr*	CTTGTGGAATGCCTTGTGATTG	AGCCGAAGCAGCACATGAT
*Mvk*	TCTGCTTGCCTTTCTCTACCTGTA	CTCGGGAGTGTCCTCTGCTT
*Mvd*	TGGTGAGCGCCGACAAG	TCTCCACGCTGGTCTGCAT
*Fpps*	ATGGAGATGGGCGAGTTCTTC	CCGACCTTTCCCGTCACA
*Srebp2*	GCGTTCTGGAGACCATGGA	ACAAAGTTGCTCTGAAAACAAATCA
*Pfkl*	CCTACGTCTTTGAGGACCCTTT	TCTGTCATATGCTCCACATTGG
*Aldob*	CTGTGTTGAGGATTGCTGACCAG	TCAGGAAGCACCTCTGGCTCAA
*Tpi1*	GGCAACTGGAAGATGAACGGGA	CTGGCAAAGTCGATGTAAGCGG
*Pgk1*	GGAAGCGGGTCGTGATGA	GCCTTGATCCTTTGGTTGTTTG
*Pkm*	GCTGTTTGAAGAGCTTGTGC	TTATAAGAGGCCTCCACGCT
*Pklr*	AGATGCAACATGCGATTGCC	GCACAGCACTTGAAGGAAGC
*Ho-1*	AAGCCGAGAATGCTGAGTTCA	GCCGTGTAGATATGGTACAAGGA
*Pgc-1a*	TCAAGCCAAACCAACAACTTTATCT	GGTTCGCTCAATAGTCTTGTTCTCA
*Pepck*	CACCATCACCTCCTGGAAGA	GGGTGCAGAATCTCGAGTTG
*G6pase*	CGACTCGCTATCTCCAAGTGA	GTTGAACCAGTCTCCGACCA
*Gapdh*	CAAGGTCATCCATGACAACTTTG	GGCCATCCACAGTCTTCTGG

## Statistical analysis

All data were evaluated using the GraphPad software (San Diego, CA, U.S.A.) and are expressed as the mean ± S.E.M. Comparisons of mean values between two groups were conducted using an unpaired two-tailed Student’s *t*-test, and multiple variable comparisons were performed using a one-way ANOVA with Tukey post hoc test. Differences were considered statistically significant at *P*<0.05.

## Supplementary material

Online supplementary figure 1

Online supplementary figure 2

Online supplementary figure 3

Online supplementary figure 4

Online supplementary table 1

Uncited online supplementary material 1

## Data Availability

All data relevant to the study are included in the article or as supplementary information. Upon reasonable request, additional information (e.g., protocols) will be shared by the corresponding authors.

## Open Access

This article has been published open access under our Subscribe to Open programme, made possible through the support of our subscribing institutions, learn more here: https://portlandpress.com/pages/open_access_options_and_prices#conditional

## Abbreviations

7-AAD: 7-aminoactinomycin D
ABC: ammonium bicarbonate
AML12: *Mus musculus* hepatocyte
DCFDA: 2,7-dichlorofluoroscin diacetate
2-DG: 2-deoxyglucose–glucose
DNL: *de novo* lipogenesis
ECAR: extracellular acidification rate
ER: estrogen receptor
ERα: estrogen receptor alpha
FACS: fluorescence-activated cell sorting
FAS: fatty acid synthase
FITC: fluorescein isothiocyanate
FITC: fluorescein isothiocyanate
GSH: reduced glutathione
GSSG: oxidized glutathione
IPA: Ingenuity Pathway Analysis
MAFLD: metabolic-associated fatty liver disease
MVA: mevalonate
25OHC: 25-hydroxycholesterol
27-OHC: 27-Hydroxycholesterol
PA/OA: palmitic/oleic acid
PBS: phosphate-buffered saline
PHD: prolyl hydroxylase domain
ROS: reactive oxygen species
SCAP: SREBP cleavage-activating protein
SERM: selective estrogen receptor modulator
24SOHC: 24S-hydroxycholesterol
TAG: triglyceride
