# Supplementary material for: Proteomic analysis reveals inhibition of mevalonate and glycolysis pathways in hepatocytes by 27-hydroxycholesterol
Source: Biochem J. 2025 Aug 4;482(15):1011–28. doi: 10.1042/BCJ20253035 (PMC12409991; doi:10.1042/BCJ20253035)
Supplement: Online supplementary figure 1 [file bcj-482-15-BCJ20253035-s001.pdf]

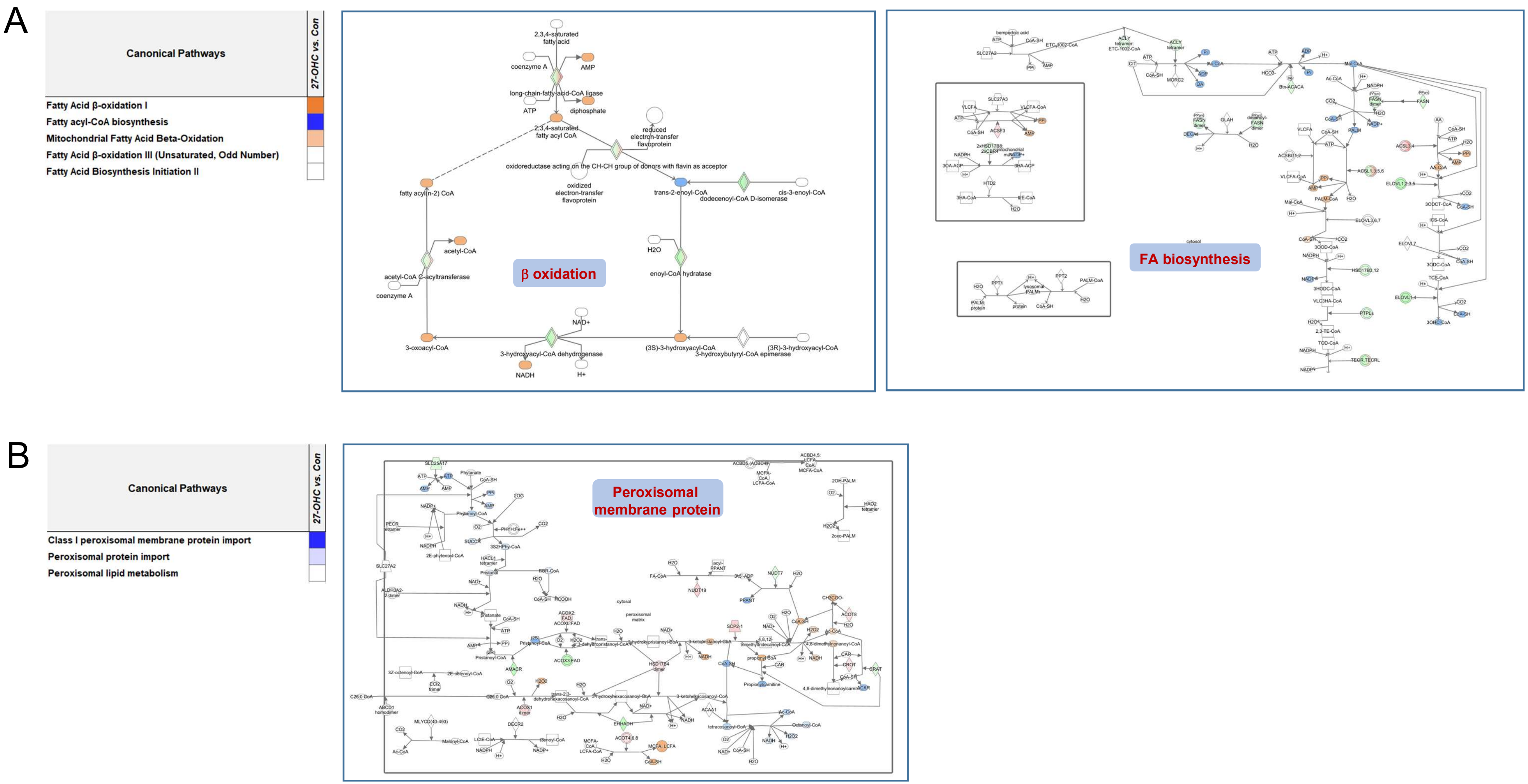

Figure 1S. Comparative canonical pathway analyses for fatty acid and peroxisome metabolism.

Comparative canonical pathway analyses using IPA. Orange and blue indicate canonical pathways with a positive or negative Z-score, respectively, reflecting 27OHC-induced pathway regulation involved in fatty acid metabolism (**A**) and peroxisome contents (**B**). n = 3 independent biological replicates.
