# Supplementary material for: Proteomic analysis reveals inhibition of mevalonate and glycolysis pathways in hepatocytes by 27-hydroxycholesterol
Source: Biochem J. 2025 Aug 4;482(15):1011–28. doi: 10.1042/BCJ20253035 (PMC12409991; doi:10.1042/BCJ20253035)
Supplement: Online supplementary figure 2 [file bcj-482-15-BCJ20253035-s002.pdf]

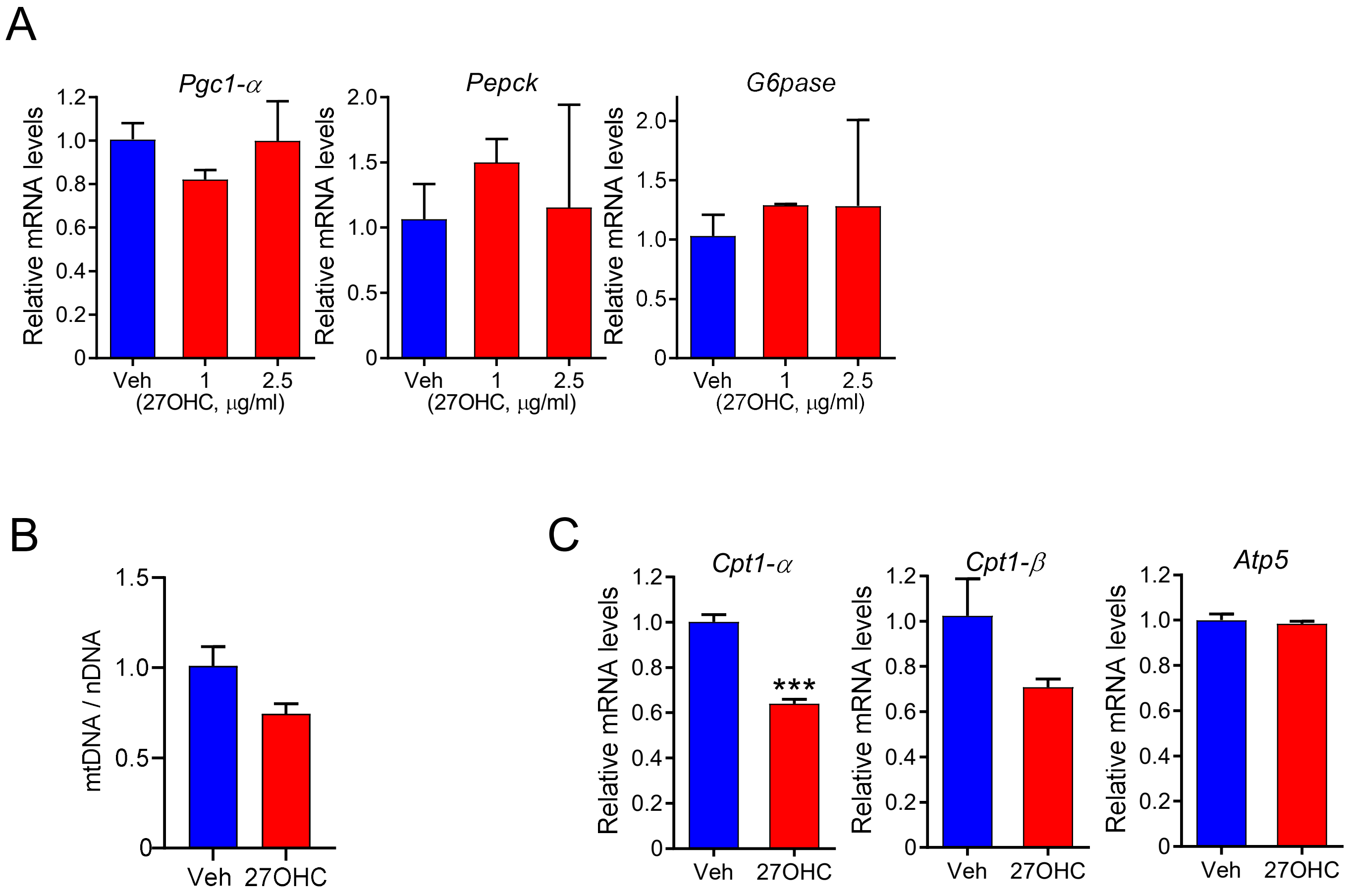

Figure 2S. Hepatic expression of rate-limiting genes involved in gluconeogenesis and mitochondrial biogenesis.

A. AML12 cells were incubated with 27-hydroxycholesterol (2.5 μg/ml) for 24 h. Relative mRNA levels of *Pgc-1a, Pepck,* and *G6pase* were determined using qPCR. **B.** Mitochondrial DNA (mtDNA) content was measured and normalized to nuclear DNA (nDNA) following 27-hydroxycholesterol (2.5 μg/ml) treatment. **C.** Expression of mitochondrial genes was measured using qPCR. n = 3 independent biological replicates.
