# Supplementary material for: Proteomic analysis reveals inhibition of mevalonate and glycolysis pathways in hepatocytes by 27-hydroxycholesterol
Source: Biochem J. 2025 Aug 4;482(15):1011–28. doi: 10.1042/BCJ20253035 (PMC12409991; doi:10.1042/BCJ20253035)
Supplement: Online supplementary figure 3 [file bcj-482-15-BCJ20253035-s003.pdf]

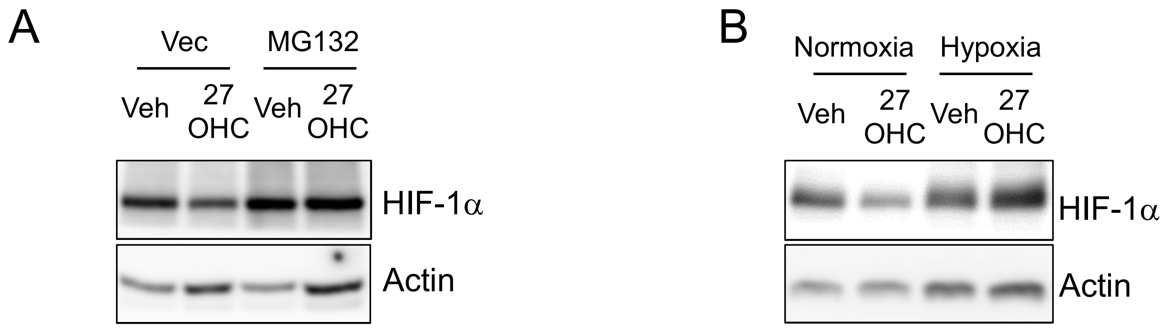

Figure 3S. MG132 treatment and hypoxic conditions prevented the inhibition of HIF-1α expression by 27OHC.

A. Effect of the proteasome inhibitor MG132 on 27-hydroxycholesterol-induced HIF-1α degradation. AML12 cells were pre-treated with the proteasome inhibitor MG132 for 3 h, then incubated with 27-hydroxycholesterol (2.5 μg/ml) for additional 6 h. HIF-1α protein levels were analyzed. **B.** The cells were incubated for 6 h with 27-hydroxycholesterol under either normoxic or hypoxic conditions to analyze HIF-1α protein levels. A representative blot is shown from three independent experiments with almost identical observations.
