# Supplementary material for: Proteomic analysis reveals inhibition of mevalonate and glycolysis pathways in hepatocytes by 27-hydroxycholesterol
Source: Biochem J. 2025 Aug 4;482(15):1011–28. doi: 10.1042/BCJ20253035 (PMC12409991; doi:10.1042/BCJ20253035)
Supplement: Online supplementary figure 4 [file bcj-482-15-BCJ20253035-s004.pdf]

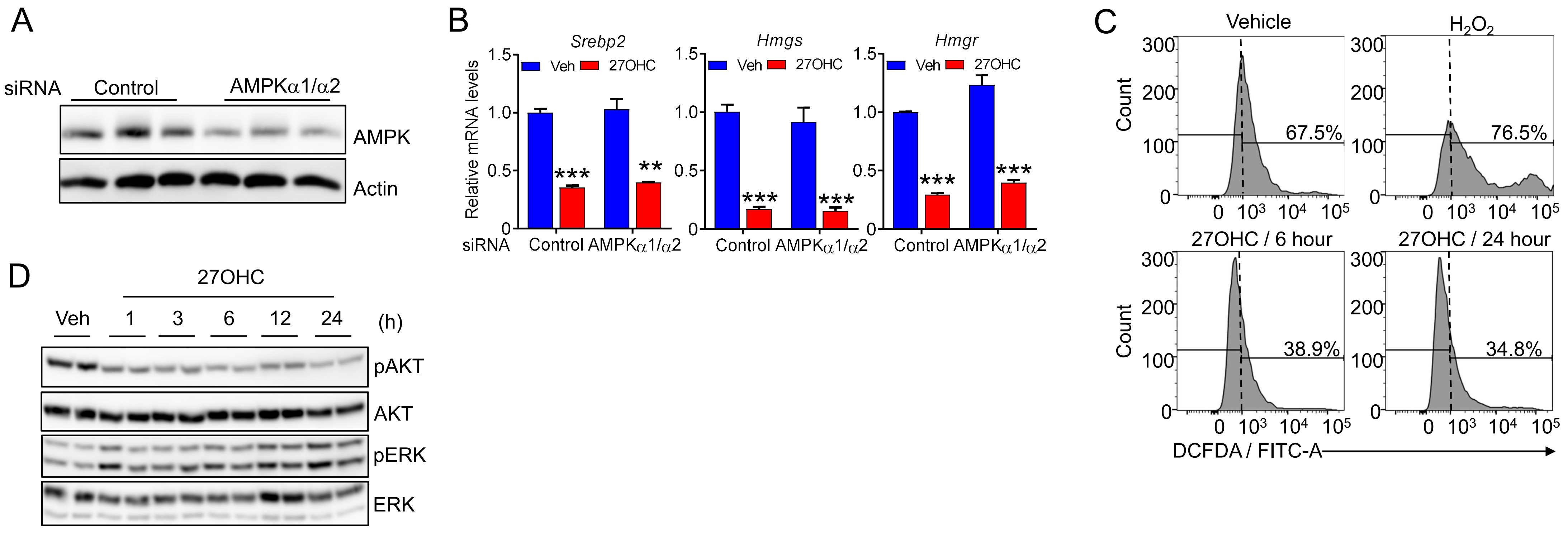

Figure 4S. Neither AMPK nor AKT pathway was involved in the effect of 27OHC.

A. AMPK knockdown efficiency in siRNA-transfected cells. AML12 cells were transfected with AMPKα1/α2 siRNA, and AMPK protein levels were measured after 24 h. **B.** After 24 h of siRNA transfection, cells were incubated with 27-hydroxycholesterol (2.5 μg/ml) for an additional 24 h. The gene expression of *Hmgs*, *Hmgr*, and *Srebp2* was measured using qPCR. Values are presented as the mean ± S.E.M. ** $p < 0.01$ and *** $p < 0.001$. **C.** Comparison of reactive oxygen species (ROS) levels after 6 and 24 h of treatment with 27-hydroxycholesterol (2.5 μg/ml). n = 3 independent biological replicates. **D.** Cells were incubated with 27-hydroxycholesterol (2.5 μg/ml) for the indicated times. Whole cell lysates were subjected to western blotting to determine the levels of phosphorylated AKT and ERK using relevant antibodies.
