# Supplementary material for: Proteomic analysis reveals inhibition of mevalonate and glycolysis pathways in hepatocytes by 27-hydroxycholesterol
Source: Biochem J. 2025 Aug 4;482(15):1011–28. doi: 10.1042/BCJ20253035 (PMC12409991; doi:10.1042/BCJ20253035)
Supplement: Uncited online supplementary material 1 [file bcj-482-15-BCJ20253035-s006.docx]

**Proteomic Analysis Reveals Inhibition of Mevalonate and Glycolysis Pathways in Hepatocytes by 27-Hydroxycholesterol Treatment**

Wan-Seog Shim^1^, Seulah Lee^2^, Bakhovuddin Azamov^1^, Chanhee Lee^1^, Yeowon Kang^1^, Kwang Min Lee^3^, Changwan Hong^4^, Sang-Mo Kwon^5^, Koanhoi Kim^6^, Dongjun Lee^1^, Jong Hyuk Yoon^2*^, and Parkyong Song^1*^

^1^Department of Convergence Medicine, Pusan National University School of Medicine, Yangsan 50612, Republic of Korea.

^2^Neurodegenerative Diseases Research Group, Korea Brain Research Institute, Daegu, 41062, Republic of Korea.

^3^Department of Life Science and Environmental Biochemistry, Pusan National University, Miryang 50463, Republic of Korea.

^4^Department of Anatomy, Pusan National University School of Medicine; Department of Convergence Medical Science, Pusan National University School of Medicine, Yangsan 50612, Republic of Korea.

^5^Laboratory for Vascular Medicine and Stem Cell Biology, Department of Physiology, Medical Research Institute, School of Medicine, Pusan National University, Yangsan 50612, Republic of Korea.

^6^Department of Pharmacology, Pusan National University School of Medicine, Yangsan, 50612, Republic of Korea.

**Supplemental figure legends**

**Figure 1S. Comparative canonical pathway analyses for fatty acid and peroxisome metabolism.**

Comparative canonical pathway analyses using IPA. Orange and blue indicate canonical pathways with a positive or negative Z-score, respectively, reflecting 27OHC-induced pathway regulation involved in fatty acid metabolism (A) and peroxisome contents (B). n = 3 independent biological replicates.

**Figure 2S. Hepatic expression of rate-limiting genes involved in gluconeogenesis and mitochondrial biogenesis.**

(A**)** AML12 cells were incubated with 27-hydroxycholesterol (2.5 g/ml) for 24 h. Relative mRNA levels of *Pgc-1α*, *Pepck*, and *G6pase* were determined using qPCR. (B) Mitochondrial DNA (mtDNA) content was measured and normalized to nuclear DNA (nDNA) following 27-hydroxycholesterol (2.5 μg/ml) treatment. (C) Expression of mitochondrial genes was measured using qPCR. n = 3 independent biological replicates.

**Figure 3S. MG132 treatment and hypoxic conditions prevented the inhibition of HIF-1α expression by 27OHC.**

(A**)** Effect of the proteasome inhibitor MG132 on 27-hydroxycholesterol-induced HIF-1a degradation. AML12 cells were pre-treated with the proteasome inhibitor MG132 for 3 h, then incubated with 27-hydroxycholesterol (2.5 g/ml) for additional 6 h. HIF-1α protein levels were analyzed. (B) The cells were incubated for 6 h with 27-hydroxycholesterol under either normoxic or hypoxic conditions to analyze HIF-1α protein levels. A representative blot is shown from three independent experiments with almost identical observations.

**Figure 4S. Neither AMPK nor AKT pathway was involved in the effect of 27OHC.**

(A**)** AMPK knockdown efficiency in siRNA-transfected cells. AML12 cells were transfected with AMPK α1/α2 siRNA, and AMPK protein levels were measured after 24 h. (B) After 24 h of siRNA transfection, cells were incubated with 27-hydroxycholesterol (2.5 g/ml) for an additional 24 h. The gene expression of *Hmgs*, *Hmgr*, and *Srebp2* was measured using qPCR. Values are presented as the mean ± S.E.M. ** p < 0.01 and *** p < 0.001. (C) Comparison of reactive oxygen species (ROS) levels after 6 and 24 h of treatment with 27-hydroxycholesterol (2.5 g/ml). n = 3 independent biological replicates. (D) Cells were incubated with 27-hydroxycholesterol (2.5 g/ml) for the indicated times. Whole cell lysates were subjected to western blotting to determine the levels of phosphorylated AKT and ERK using relevant antibodies.
